# Early ACL reconstruction shows an improved recovery of isokinetic thigh muscle strength compared to delayed or chronic cases

**DOI:** 10.1007/s00402-023-04863-5

**Published:** 2023-04-13

**Authors:** Markus Wenning, Marlene Mauch, Albrecht H. Heitner, Gerrit Bode, Ghislain Sofack, Ramona Ritzmann

**Affiliations:** 1 Rennbahnklinik, Kriegackerstr. 100, 4132 Muttenz, BL Switzerland; 2 Department of Orthopedic and Trauma Surgery, Faculty of Medicine, University Medical Center, University of Freiburg, Hugstetter Str. 55, 79106 Freiburg, Germany; 3 Praxisklinik 2000, Wirthstr. 11, 79100 Freiburg, Germany; 4 Institute of Medical Biometry and Statistics, Faculty of Medicine, University of Freiburg, Zinkmattenstr. 6a, 79108 Freiburg, Germany; 5 Institute of Sport and Sport Science, Department of Motor Control, University of Freiburg, Freiburg, Germany; 6 Department of Orthopaedics and Traumatology, University Hospital Basel, Basel, Switzerland

**Keywords:** ACL reconstruction, Return-to-sports, Isokinetic strength, Timing of surgery

## Abstract

**Introduction:**

The recovery of periarticular strength is a major criterion in return-to-play testing. The rationale of the study was to assess the impact of the delay of surgery (∆ between injury and surgery) on knee extensor and knee flexor strength of anterior cruciate ligament (ACL)-deficient patients six months after reconstruction.

**Materials and methods:**

In a retrospective cohort study, all patients with ACL ruptures between 03/2015 and 12/2019 were analyzed. Inclusion criteria were isolated ACL rupture without any associated lesions undergoing a reconstruction using ipsilateral hamstring tendon autograft and adherence to isokinetic strength testing before and at 5–7 months postoperatively. These patients were then clustered into three groups: EARLY reconstruction (∆ < 42 days), DELAYED reconstruction (∆42-180d), and CHRONIC (∆ > 180d). Knee extensor and flexor strength of the ipsi- and contralateral leg were analyzed by concentric isokinetic measurement (60°/s). Primary outcomes were the maximal knee extension and flexion torque, hamstrings-to-quadriceps ratio (H/Q) ratio), and the corresponding limb symmetry indices.

**Results:**

*n* = 444 patients met the inclusion criteria. From EARLY to DELAYED to CHRONIC, a progressive reduction in postoperative strength performance was observed in knee extension (1.65 ± 0.45 to 1.62 ± 0.52 to 1.51 ± 0.5 Nm/kg resp.) and flexion (1.22 ± 0.29 to 1.18 ± 0.3 to 1.13 ± 0.31 Nm/kg resp.) strength on the ACL reconstructed leg. This general loss in periarticular strength was already apparent in the preoperative performance even on the healthy side. When controlling for the preoperative performance using ANCOVA analysis, EARLY performed significantly better than DELAYED (extension *p* = 0.001, flexion *p* = .02) and CHRONIC (extension *p* = 0.005, flexion *p* < 0.001). Also, there were significantly higher values for H/Q ratio in the injured leg across all groups where the H/Q ratio increased from EARLY to CHRONIC and from pre- to postoperative values.

**Conclusions:**

With respect to the force generating capacity when returning-to-play, it is advantageous to seek for an early ACL reconstruction within the first 12 weeks after the injury. The increasing loss of thigh muscle strength observed in delayed or chronic cases affects the injured and also the non-injured leg.

**Level of Evidence:**

III, retrospective cohort study.

## Introduction

Ruptures of the anterior cruciate ligament (ACL) are among the most common and impactful injuries in athletes leading to an incidence of 46/100.000 ACL-reconstructions per year in Germany [1]. It has been widely shown that the ACL-deficient knee will enter into an early degenerative process, including accelerated meniscal and cartilage damage [2]. This led to the consensus of timely surgical stabilization to maintain arthrokinematics in the long run [3]. Still, there is no established time frame in which it is deemed optimal to perform surgery [4, 5]. Furthermore, the odds of successfully repairing a meniscal lesion decrease over time, while the prevalence of cartilage injury increases [5, 6]. Moreover, some authors advocate that long-term subjective and objective outcomes are negatively affected by delayed surgery [5–7]. Recent systematic reviews indicate that there is no significant difference in joint function between early (within the first 6 weeks) and delayed surgery (until up to 6 months) [8–10].

Contrarily, the incidence of postoperative arthrofibrosis has been the main reason to delay surgery until the end of the inflammatory phase [11]. However, the rate of range-of-motion (ROM)-deficits has been very low since the adoption of progressive functional rehabilitation [9]. Next, the recovery of preoperative function could be a benefit worthy of delaying surgery and the evidence for pre-habilitation is ever increasing [10–12]. Earlier studies indicated that immediate reconstruction results in slower recovery, even if accelerated rehabilitation schemes were used [11]. Since functional performance is reduced on the injured side instantaneous after the injury, neuro-mechanical processes may persistently inhibit the postoperative recovery even if the ligament is reconstructed instantaneously [13].

Only few studies describe the general course of athletic performance following delayed surgery. It is believed that the contralateral leg maintains its strength for a longer period of time [14, 15], while the injured leg may recover within about 12 weeks following the trauma [5, 9, 11]. Evidently, for most of the physically active patients and elite athletes, three months is not a desirable time to wait for surgery. The reduction in athletic activity and ability will cause a loss in general performance, consequently also the non-injured side may be affected negatively, while waiting for ACL reconstruction [16, 17].

Summarizing this dilemma between the mechanical and structural disadvantages and the recovery of preoperative function, it is of high scientific and clinical interest whether strength performance will also be affected by the timing of surgery. Despite the numerous publications on thigh muscle strength following ACL reconstruction, the evidence on that effect is scarce. Nonetheless, when considering the established requirements of an overall symmetric performance before returning to play, it is of high interest which timing for ACL- reconstruction leads to superior postoperative performance [18].

Therefore, the purpose of the present study is to assess the impact of surgical delay (∆ between injury and surgery) on knee extensor and flexor strength six months after ACL reconstruction. We hypothesized that a longer duration between injury and surgery will lead to a better recovery of muscular function, while the overall strength performance will be reduced [14, 17, 19– 21].

## Methods

We performed a retrospective analysis of all patients in the database of our performance laboratory which were undergoing primary arthroscopic ACL reconstruction between March 1, 2015 and December 31, 2019. The dataset has been progressively increased to collect the results of all performance testing in this institution following ACL reconstruction.

### Study population

Inclusion criteria were primary ACL reconstruction using hamstring autograft and the participation in pre- and postoperative isokinetic strength testing. Exclusion criteria were associated injuries with a direct impact on postoperative rehabilitation, such as meniscal repair, concomitant ligament injury, cartilage intervention, revision surgery, medial collateral ligament injury, etc. Exclusion criteria were verified peri-operatively according to MRI and arthroscopic findings as well as intraoperative procedures. All surgeons (seven in total) were experienced orthopedic surgeons with an average of 5–17 years of experience in arthroscopic knee surgery. The intraoperative proceedings were standardized according to the current state-of-the art using an anteromedial portal for femoral drilling, proximal fixation using an endo-button and an interference screw for tibial fixation. The study cohort was subdivided into three groups: group 1 had an EARLY reconstruction with a time interval between injury and surgery < 42 days. Group 2 had a DELAYED reconstruction 42–180 days after the injury. And group 3 had a reconstruction of CHRONIC tears > 180 days [10]. The sports performance was classified according to Grindem et al. [22] as follows: (1) sedentary/no sports, (2) low-volume athletic activity (3) straight ahead sports (i.e., running, cross-country skiing, cycling) (4) pivoting sports (i.e., soccer, American football, skiing, combat sports) [22].

### Postoperative rehabilitation

The postoperative treatment was identical for all patients, which followed a criterion-based rehabilitation including mono-articular exercises and passive treatment for 2–4 weeks, while full weight-bearing was allowed as soon as there were no signs of inflammation or effusion/pain, which was generally achieved within the first two weeks. Patients then increased their physical activity stepwise aiming to achieve symmetrical gait at 6 weeks the latest. The first postoperative strength measurement was an isometric (submaximal) test, y-balance test, and balance squat at 12 weeks before allowing a return to straight running, if limb symmetry was acceptable (> 85%). The isokinetic strength testing was performed around 6 months postoperatively, if the patient had been able to systematically follow the rehabilitation criteria, which was generally achieved by the patients. Those patients performing their rehabilitation at different institutions were required to undergo testing by an in-house physiotherapist before isokinetic strength testing and being cleared for returning to play. If the isokinetic strength testing was successfully passed, a stepwise return to sport-specific tasks including cutting and jumping was allowed.

### Isokinetic strength testing

**Fig. 1:**
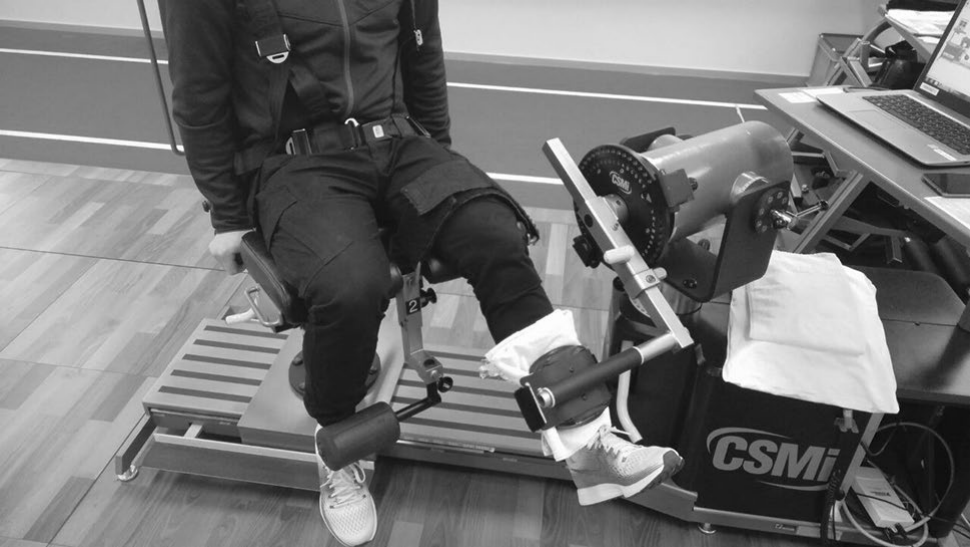
Setup for isokinetic strength testing

The isokinetic strength testing was performed as previously described with the assessment of knee extensor and flexor strength using an isokinetic dynamometer (Humac®/NormTM Testing & Rehabilitation System, Computer Sports Medicine, Inc. (CSMi, Stoughton, Massachusetts, US) according to Li and Wu [23], see Fig. 1. The isokinetic testing evaluator was blinded to the assigned group in this retrospective analysis.

Each subject was sitting upright, the trunk at approximately 100° leaning against the backrest of the testing table, fixed by straps across the chest and a horizontal pad over the middle third and proximal half of the distal third of the thighs. The knee joint axis was aligned with the mechanical axis of the dynamometer. The shin pad was placed just superior to the medial malleolus.

Prior to each test sequence, subjects performed a standardized 10 min warm-up on a cycling ergometer (50W) followed by three submaximal repetitions to familiarize with the testing procedure. For data assessment, we use concentric–concentric contractions at 60°/s angular speed, in the full individual range of motion (ROM) due to its high test–retest reliability [24]. Two sets of three repetitions with maximum effort were executed with a resting time of 1 in between the sets. Each trial was initiated with the unaffected limb.

Primary outcome parameters were: maximal knee extension and flexion torque normalized to body mass ([Nm/kg]), the hamstrings-to-quadriceps ratio (H/Q ratio), and the limb symmetry index (affected limb/unaffected limb*100) for the knee extensors and flexors. For data assessment, the better of the two sets was selected.

### Statistics

All statistical analyses were run as complete case analyses. Descriptive statistics were expressed as means and standard deviations. The primary independent variable was the timing of surgery subgroups. Prior to statistical analyses, baseline pre-operative characteristics of patients, namely age, sex, and body mass index, were compared between the three timing periods of surgery subgroups, and no statistically significant differences were found; hence, no matching was done. A one-way analysis of variance (ANOVA) test was used to determine the effect of timing of surgery on the postoperative flexion and extension forces. In addition, an analysis of covariance (ANCOVA) test was performed with adjustment for the preoperative forces, to determine the effect of timing of surgery on the postoperative strength. An independent sample t test was used to determine the mean difference in H/Q ratio between the injured and non-injured leg. All assumptions for independent samples, Student’s t tests, ANOVA, and ANCOVA were tested and fulfilled. The presence of normal distributions and the number of outliers in outcomes were checked using data exploration techniques. The normality of the residuals was confirmed using the Shapiro–Wilk test, and homogeneity of variance was present as the Levene’s test suggested. The level of significance was defined at *p* < 0.05, and significant ANCOVA results were further explored using Bonferroni post hoc comparison tests. Effect sizes were labeled following Field's (2013) recommendations. Statistical analysis was conducted using R (R v. 4.1.2). Graphical display was performed using Veusz (Veusz v. 3.0.1 by J. Sanders et al.).

## Results

**Fig. 2:**
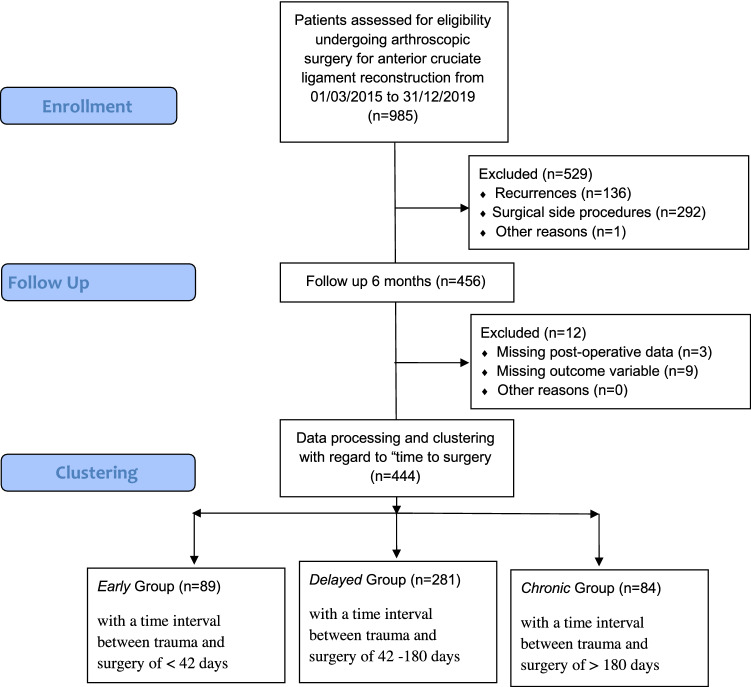
Study flow chart diagram

**Fig. 3:**
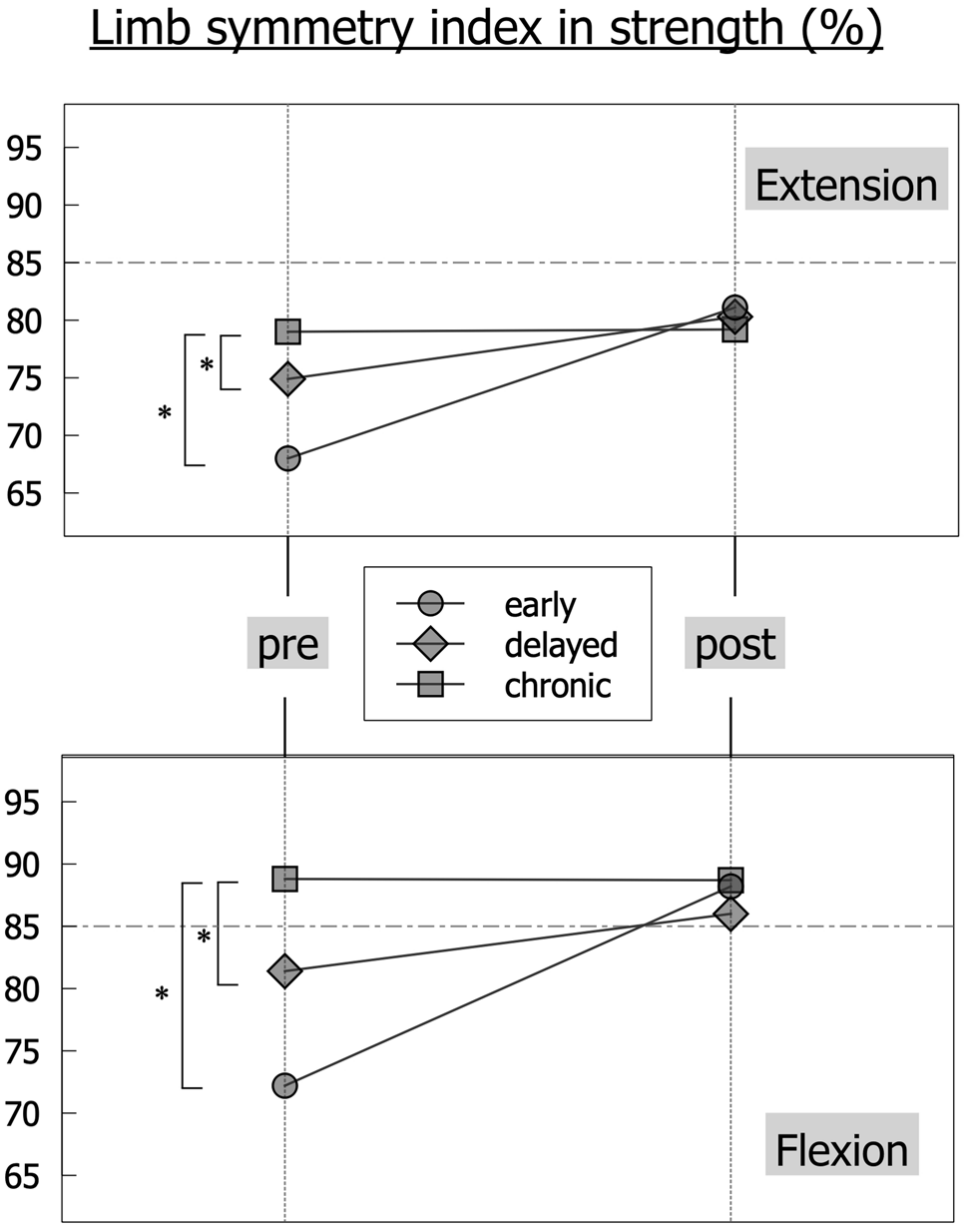
Limb symmetry indices across the groups from pre- to postoperative values, * = *p* < 0.05, for standard deviations, see Tables 2 and 3

**Fig. 4:**
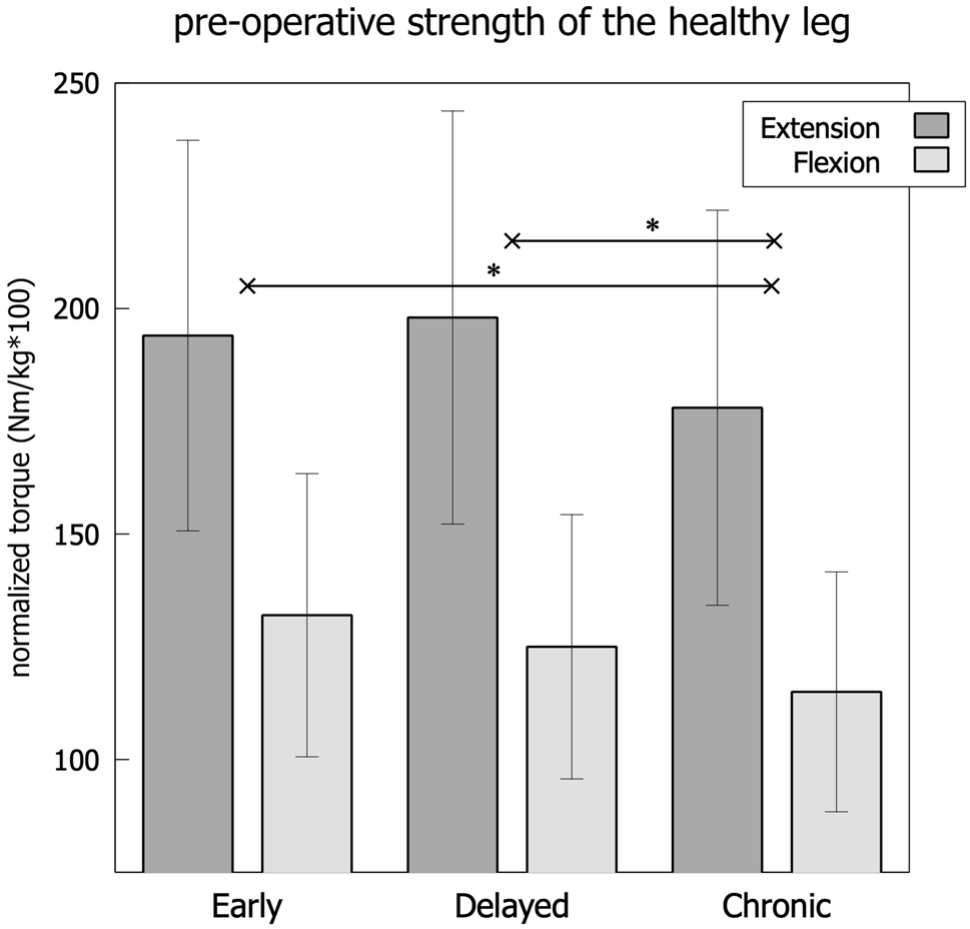
Pre-operative isokinetic strength of the healthy leg across the groups. *Referring to the limb symmetry index and significant differences at *p* < 0.05

**Fig. 5:**
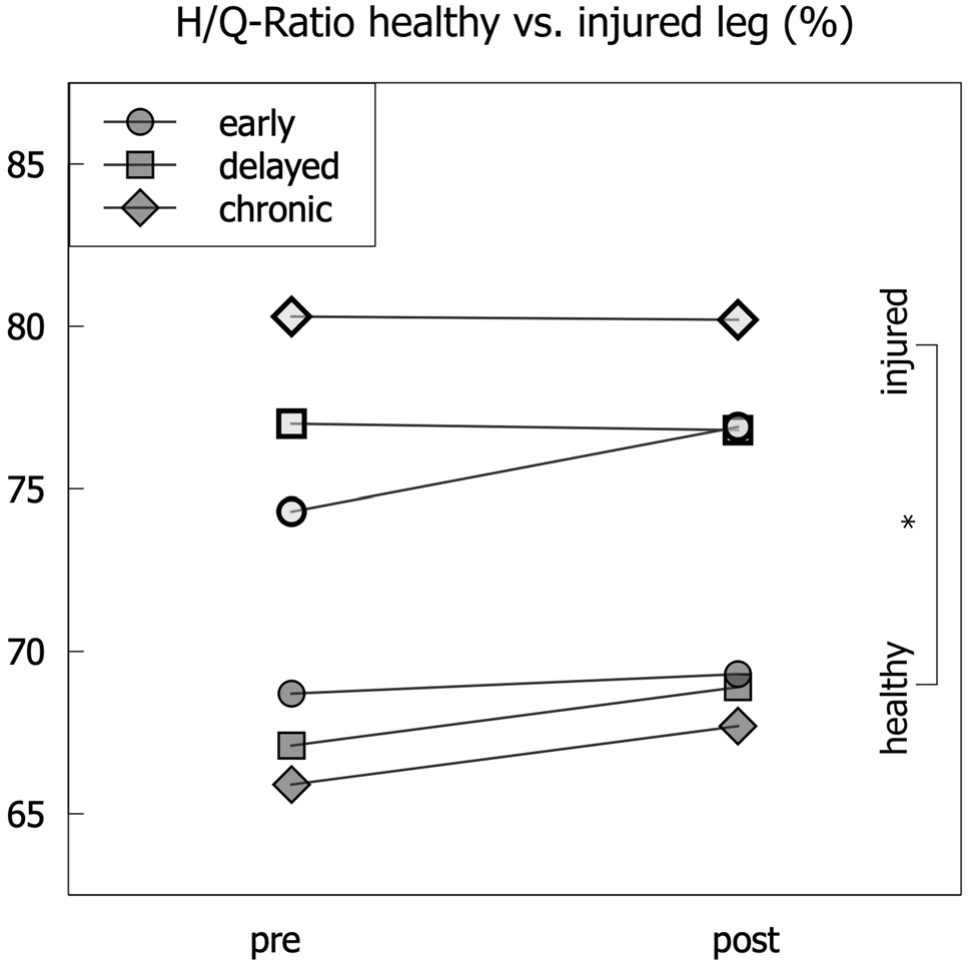
H/Q ratio across the groups and change from pre- to postoperative values * = *p* < 0.05, for standard deviations, see Tables 2 and 3

**Fig. 6:**
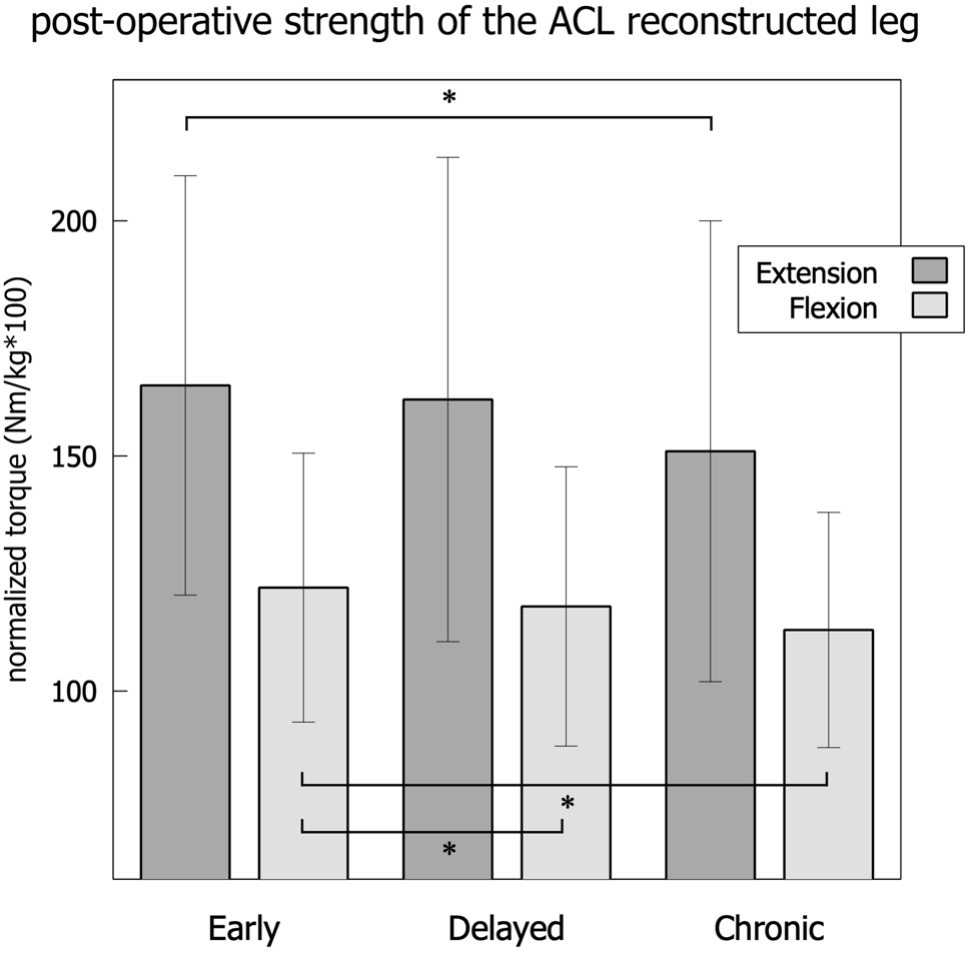
Post-operative isokinetic strength of the ACL reconstructed leg across the groups, * = *p* < 0.05

A total of 444 out of 985 patients undergoing ACL reconstruction could be included in this retrospective cohort study. The composition of the subgroups is listed in Table 1. 541 patients had to be excluded due to exclusion criteria and missing data (see Fig. 2). Table 1 shows sex, age, and anthropometric data of the three subgroups. There were no significant differences in any of the factors including age (*p* = 0.79), sex (*p* = 0.8), and anthropometric measures (*p* = 0.13). The “EARLY” group resulted in *n* = 89 patients undergoing surgery at a median of 31 days post-injury, the “DELAYED” group of *n* = 271 patients undergoing surgery at a median of 78 days post-injury and a “CHRONIC” group of *n* = 84 patients undergoing surgery at a median of 344 days after the initial injury. The results of the study are summarized in Figs 3, 4, 5, 6, while Tables 2 and 3 carry the detailed numbers and absolute strength values of our findings.

**Table 1:** Biometrical composition of the subgroups

	Early		Delayed		Chronic	
Number (*n*)	89		271		84	
∆ Injury to surgery *(in d, median (IQR)*	*31 *(17)		78 (51)		344 (652)	
Height (in m, *Mean (SD)*)	1.76 (0.09)		1.75 (0.09)		1.74 (0.08)	
Weight (in kg, *Mean (SD)*)	75.2 (13.0)		76.4 (13.2)		75.9 (12.6)	
BMI (*Median (IQR)*)	24.1 (3.8)		24.3 (4.2)		24.5 (4.1)	
Age at surgery (in y, *Median (IQR)*)	25.3 (14)		26.8 (13)		27.4 (18)	
Age according to subgroup	*n*	*%*	n	%	*n*	%
10–19	14	*16*	43	16	19	23
20–29	44	*50*	116	43	27	32
30–39	15	*17*	64	24	17	20
40–49	10	*11*	36	13	18	21
> 50	6	*7*	12	4	3	4
*Sex*						
Male	64	*72*	194	72	57	68
Female	25	*28*	77	28	27	32
Dominant leg injured	54	*61*	153	56	54	64
*Sports classification* [22]						
1	6	7	3	1	10	12
2	5	6	30	11	9	11
3	22	24	68	25	18	21
4	56	63	170	63	47	56

**Table 2:** Preoperative isokinetic strength according to the subgroup

Preoperative values		Extension	LSI Ex	Flexion	LSI Flex	H/Q ratio
		Mean ± SD (Nm/kg)	Mean ± SD (%)	Mean ± SD (Nm/kg)	Mean ± SD (%)	Mean ± SD
Early	OP	*1.31* ± *0.53*^*#*^	*68* ± *22.6*^+^	*0.95* ± *0.38*^*#*^	*72.2* ± *23.8*^+^	*74.3* ± *16.3*^*#*^
*n* = 89	NOP	*1.94* ± *0.43*^+^		*1.32* ± *0.31*^+^		68.7 ± 10.6
Delayed	OP	*1.43* ± *0.57*^*#*^	*74.9* ± *23.9*^+^	*1.02* ± *0.37*^*#*^	*81.4* ± *24.0*^+^	*77.0* ± *29.2*^*#*^
*n* = 271	NOP	*1.98* ± *0.46*^+^		*1.25* ± *0.3*^+^		67.1 ± 11.6
Chronic	OP	*1.4* ± *0.52*^*#*^	*79.0* ± *23.8*	*1.02* ± *0.32*^*#*^	*88.8* ± *22.3*	*80.3* ± *41.2*^*#*^
*n* = 84	NOP	*1.78* ± *0.44*		*1.15* ± *0.27*		65.9 ± 10.4

*OP *operated*, NOP *not affected leg*, Nm *newton meter*, LSI *limb symmetry index*, Ex *Extension strength*, Flex *flexion strength*, SD *standard deviation

^+^significant between-groups difference compared to chronic group: ^+^*p* < 0.05, ^++^*p* < 0.01)

^#^significant within-group difference compared to healthy leg (*p* < 0.05)

**Table 3:** Postoperative isokinetic strength according to the subgroup

Postoperative values		Extension	LSI Ex	Flexion	LSI Flex	H/Q ratio
		Mean ± SD (Nm/kg)	Mean ± SD (%)	Mean ± SD (Nm/kg)	Mean ± SD (%)	Mean ± SD
Early	OP	*1.65* ± *0.45*^*#*+^	*81.1* ± *15.4*	*1.22* ± -29^*#^	*88.2* ± *13.1*	*76.9* ± *17.0*^*#*^
*n* = 89	NOP	*2.04* ± *0.45*^+^		*1.40* ± 0.3^*^		69.3 ± 9.75
Delayed	OP	*1.62* ± *0.52*^*#*^	*80.3* ± *19.0*	*1.18* ± *0.3*^*#*^	*86.0* ± *13.8*	*76.8* ± *21.0*^*#*^
*n* = 271	NOP	*2.02* ± *0.44*		*1.37* ± *0.28*		68.9 ± 10.5
Chronic	OP	*1.51* ± *0.49*^*#*^	*79.2* ± *19.0*	*1.13* ± *0.3*^*#*^	*88.7* ± *14.0*	*80.2* ± *27.7*^*#*^
*n* = 84	NOP	*1.91* ± *0.46*		*1.27* ± *0.3*		67.7 ± 11.1

*OP *operated,* NOP *not affected leg,* Nm *newton meter,* LSI *limb symmetry index,* Ex *Extension strength,* Flex *flexion strength,* SD *standard deviation

^+^significant difference between groups compared to chronic group in ANCOVA, *p* < 0.05

^*^significant difference between groups compared to delayed and chronic group in ANCOVA, *p* < 0.05

^#^significant difference within group compared to healthy leg

### Preoperative values

There were significant differences between the groups in preoperative limb symmetry in extension and flexion strength (Fig. 3, ANOVA *p* < 0.05). For the extension strength, the CHRONIC group showed the highest limb symmetry index (LSI) (79.0%), followed by the DELAYED (74.9%) and the EARLY (68%) group (Fig. 3). The mean bodyweight-normalized values were the lowest in EARLY reconstruction (1.31 Nm/kg), followed by the CHRONIC group (1.4 Nm/kg) and the DELAYED group (1.43 Nm/kg). Of note, the performance of the non-injured leg was significantly lower in the CHRONIC group (1.78 Nm/kg) when compared to the EARLY (1.94 Nm/kg) and DELAYED (1.89Nm/kg) group (*p* = 0.04, Fig. 4 and Table 2). The values of flexion strength followed a comparable pattern as displayed in Table 2. The values for H/Q ratio showed a significantly higher H/Q ratio (*p* < 0.001, Fig. 5) in the injured leg compared to the non-injured leg within each group. The between-groups comparison showed increasing H/Q ratios from EARLY to CHRONIC reconstruction on the injured side and decreasing H/Q ratios on the non-injured side.

### Postoperative values

After adjustment for preoperative extension forces implementing a fitting ANCOVA, there was a statistically significant effect of the timing of surgery on postoperative extension strength (F(2,440) = 5.73, *p *= 0.003, Fig. 6). Post hoc analysis using Bonferroni adjustment showed that the EARLY group, was significantly different from the CHRONIC group (*p* = 0.002). There were no statistically significant differences between the EARLY group and the DELAYED group (*p* = 0.0992), and neither between the DELAYED group and the CHRONIC group (*p* = 0.130). There was also a statistically significant difference in postoperative flexion forces between the groups in ANCOVA analysis, implementing preoperative strength as a factor (F(2,440) = 6.81, *p* = 0.012, Fig. 6). Post hoc analysis using Bonferroni adjustment showed that the EARLY group, was significantly different from the DELAYED group (*p* = 0.02) and also from the CHRONIC group (*p* < 0.001). No statistically significant difference was found between the DELAYED group and the CHRONIC group (*p* = 0.259).

For the H/Q ratio at 6 months postoperatively, the values of the injured leg remained significantly higher than the non-injured leg within the group itself. For a between-groups comparison, the largest side-to-side difference (injured vs. non-injured leg) in H/Q ratio was observed for the CHRONIC group (12.5%), followed by the DELAYED group (7.9%) and the EARLY group (7.6%). However, this deficit was not significantly different between the groups.

## Discussion

The main finding of the present study is that post-operative thigh muscle strength is significantly reduced if the reconstruction is delayed for more than six months after the injury. Contrarily, bilateral pre-operative strength performance is more symmetric in delayed cases due to an overall decrease in strength, which affects the injured as well as the non-injured leg.

Taking into account the functional adaptations observed in this study including patients’ performance and sighting a safe return-to-play, patients will benefit from timely surgical reconstruction [5, 8, 25, 26].

### Optimal timing of surgery

To date, there is no consensus on the optimal timing of surgical reconstruction [27, 28]. Some authors advocate awaiting the end of the inflammatory phase, which is supposed to end around 3–6 weeks post-injury. However, postoperative arthrofibrosis due to increased capsular inflammation etc., is very rare if rehabilitation schemes are progressive [29, 30]. Another reason to delay surgical reconstruction is the most current concept of pre-habilitation, i.e., optimizing periarticular function by supervised passive and active therapy before surgery. In this regard, the available evidence suggests that a 5-week (Grindem et al. [26]) or 6-week intervention (Shaarani et al. [29]) will lead to superior postoperative performance, range-of-motion and patient satisfaction [13, 25].

Contrarily, there are also authors underscoring an early stabilization of the joint: there is good evidence by Fithian et al. that late reconstruction will lead to more meniscal injuries [9, 11]. The authors defined “late” as any reconstruction later than 12 weeks after injury. Comparable results were found in an adolescent cohort by Lawrence et al., who observed that time to surgery is a risk factor for irreparable meniscal lesions and an increase in chondral lesions especially in subjectively unstable patients [27]. Lawrence et al. also defined “late” as anyone undergoing surgery > 12 weeks after injury [27].

The effect that the timing of surgery will have on postoperative strength performance is important when managing patients’ expectations on postoperative athletic performance and return-to-play [15, 31, 32]. Especially if patients aim to recover to their preoperative level of performance, a reconstruction should be performed at least within the first six months after injury [5, 8]. Moreover, it is important to mention that a delayed ACL reconstruction will also require more time to safely progress through rehabilitation. This will lead to an additional delay in returning-to-play [12, 33, 34]. The data presented in this study shows several aspects reflecting the decrease of thigh muscle strength. Since there is a strong correlation between isokinetic strength deficits and patient-reported outcome [35], this yields that early ACL reconstruction is likely to increase long-term patient satisfaction. With regard to the minimal clinically important difference, the differences in strength between the groups in this study show a relative change from EARLY to CHRONIC of 8–9%, which signifies a relevant impact [36].

Recapitulating these findings, we may suggest that the optimal timeframe for reconstruction of the ACL is < 12 weeks after the injury while the time from injury to surgery should be used for pre-habilitation.

### Extension strength

While the EARLY group enters surgery with a highly imbalanced extension strength, it overall achieves the best limb symmetry six months after surgery. Moreover, the non-injured leg is likely to improve its strength performance above the pre-injury level since the postoperative rehabilitation program will affect both legs. Contrarily, the CHRONIC group has generally recovered limb symmetry before surgery (LSI 79% in extension and 88% in flexion); still, this is mainly the consequence of an overall loss of strength in the non-injured leg. This finding underscores that it is not only the intra-articular damage caused by the instability, but also periarticular changes like a reduction in general strength that affect the athletic performance negatively. This effect seems to persist for longer than six months despite postoperative rehabilitation. In summary, the recovery of knee extension strength in the reconstructed and the non-injured leg requires more time in chronic patients compared to early reconstruction. Future studies need to evaluate the causes for this persisting performance deficit. Potentially, the reduction of overall athletic activity and/or the adoption of a different neuromuscular activation pattern due to the long-term instability may play a role in this. Future analysis may also elucidate, if the observed loss of thigh muscle strength of the contralateral “healthy” leg can explain the increased risk of subsequent contralateral ACL rupture.

### Flexion strength

Interestingly, flexion strength is not affected in a comparable manner as the extension strength (higher LSI values). Secondary, this leads to higher H/Q ratios on the injured side. Factors like pain and swelling will most likely reduce preoperative performance, while neuro-mechanical factors, like arthrogenic inhibition may account for prolonged postoperative weakness [36–38]. In the present study, all patients had received hamstring autografts, therefore this observation is not a matter of the graft site. However, it will require additional research to see whether this observation is the case in other grafts as well. Effectively, it may be even more distinct in grafts of the knee extensor unit.

The fact that the outcome in the CHRONIC group has the highest pre- and postoperative H/Q ratios on the injured side, may also yield a neuromuscular adaptation when considering the hamstrings’ function in limiting anterior tibial translation [39]. The ipsilateral increase in H/Q ratio could be an indicator of a functional adaptation to actively prevent excessive anterior translation of the tibia. Future research should clarify, if the relative increase in hamstring strength reflects a tendency toward hamstring-quadriceps co-contraction for actively stabilizing the ACL-deficient joint. This pattern may be adopted in a more stable way by patients of the chronic group and thus persist despite surgical stabilization.

### Definition of time frames

For the purpose of this study, the definitions of the different time phases were framed following clinical and scientific appearance: EARLY reconstruction was intended to include those patient undergoing reconstruction within the inflammatory phase, DELAYED reconstruction after the end of the inflammatory phase [25]. The patients in the DELAYED group may therefore show a more robust performance compared to EARLY, since peri- and intra-articular factors limiting neuromuscular activation may have disappeared. Since the definition of chronic cases for any patient reconstructed > 6 months after injury is widely accepted, we adopted this time point for the CHRONIC cases [8, 9, 29].

### Limitations

The limitations of this study are mainly due to its retrospective nature: the preoperative level of performance was not reported sufficiently, which may have resulted in a recruiting bias, such that the chronic group might have been more prone to delaying surgery due to a lower level of athletic ambition. However, since we mainly used intra-individual values or corrected for intra-individual changes, we may assume that this did not affect the main findings of this study. Moreover, even though there was a significant difference in the classification of sports (*p* < 0.001) performed in each group, this difference seems negligible when looking at the percentages of participants in each type of sports across the groups. Another limitation is the limited information on the treatment between injury and surgery, which was not systematically monitored in this study group and thus, it was not feasible to assess its potential influence.

The strengths of the study include the large sample size and the homogeneity of the treating algorithm as well as the objective analysis of strength. All patients were treated by the same surgical algorithm. Moreover, we chose to include isolated ACL repairs only in order to create even more homogenous cohorts [40, 41].

## Conclusions

An early ACL reconstruction surgery is advantageous compared to a delayed and especially a chronic reconstruction. Delay of surgery affects the force generating capacity of the musculature encompassing the knee joint on the injured and also on the non-injured leg.

## Data Availability

Data will be made available by the corresponding author upon reasonable request.
